# The usability of ark clam shell (*Anadara granosa*) as calcium precursor to produce hydroxyapatite nanoparticle via wet chemical precipitate method in various sintering temperature

**DOI:** 10.1186/s40064-016-2824-y

**Published:** 2016-07-29

**Authors:** Mohammad Zulhasif Ahmad Khiri, Khamirul Amin Matori, Norhazlin Zainuddin, Che Azurahanim Che Abdullah, Zarifah Nadakkavil Alassan, Nur Fadilah Baharuddin, Mohd Hafiz Mohd Zaid

**Affiliations:** 1 Material Synthesis and Characterization Laboratory, Institute of Advanced Technology, Universiti Putra Malaysia (UPM), 43400 Serdang, Selangor Malaysia; 2 Department of Physics, Faculty of Science, Universiti Putra Malaysia (UPM), 43400 Serdang, Selangor Malaysia; 3 Department of Chemistry, Faculty of Science, Universiti Putra Malaysia (UPM), 43400 Serdang, Selangor Malaysia

**Keywords:** Hydroxyapatite, Ark clam shell, Wet chemical precipitate, Sintering, Structural

## Abstract

This paper reported the uses of ark clam shell calcium precursor in order to form hydroxyapatite (HA) via the wet chemical precipitation method. The main objective of this research is to acquire better understanding regarding the effect of sintering temperature in the fabrication of HA. Throughout experiment, the ratio of Ca:P were constantly controlled, between 1.67 and 2.00. The formation of HA at these ratio was confirmed by means of energy-dispersive X-ray spectroscopy analysis. In addition, the effect of sintering temperature on the formation of HA was observed using X-ray diffraction analysis, while the structural and morphology was determined by means of field emission scanning electron microscopy. The formation of HA nanoparticle was recorded (~35–69 nm) in the form of as-synthesize HA powder. The bonding compound appeared in the formation of HA was carried out using Fourier transform infrared spectroscopy such as biomaterials that are expected to find potential applications in orthopedic and biomedical industries .

## Background

Recently, hydroxyapatite (HA) was widely used in the field of orthopedic and biomedical application (Rujitanapanich et al. 2014; Singh and Purohit 2011; Dědourková et al. 2012; Hoque et al. 2014). The structure of HA which is similar to the structure of bone become the main criteria in the creation and innovation of future synthetic bone. HA has been synthesis using various methods, and the most practical technique is known as wet chemical precipitation. HA with the molecular formula of Ca_10_(PO_4_)_6_(OH)_2_ is one of the essential mineral consist from the calcium phosphate salt group (Rajkumar et al. 2011). It is the most stable calcium phosphate salt among the other salt such as tricalcium phosphate (TCP) and tetracalcium phosphate (TTCP) at the range pH value in between 4 and 12 and at normal temperatures (Koutsopoulos 2002; Sadat-Shojai et al. 2013; Abidi and Murtaza 2014). The composition of HA will be formed when the stoichiometric of Ca/P ratio approximately in the range of 1.67 (Rujitanapanich et al. 2014; Abidi and Murtaza 2014). HA is usually found in the vertebrate group, especially in the hard tissue of bone and teeth enamel. The similarity properties of the chemical composition and the crystal structure of HA with the natural human bone give an alternative biomedical application uses in the human body such as bone implant for bone tissue substitution and drug delivery system (Santhosh and Prabu 2013). Besides, HA can be directly ingrowths in the host bone as artificial bones in human body and it was widespread used in orthopedic application due to its special abilities in bioactivity and biocompatibility (Zhang 2007).

HA is not only biocompatible, nontoxic, esteoconductive, non-inflammatory, but also bioactive (Fathi et al. 2008). The biocompatibility refer to the properties of material which is show biologically compatible with living system without gives any respond in local or systemic environment while bioactivity can be describe in any interaction or effect of material on living system. These properties are very important for synthetic bone which acts as bone grafting in successfully of osteoconductivity. This compound was commonly used as coating on metal implant to enhance their osteointegration (ability of biomaterial that can guide the reparative growth of natural bone) which promote the bone ingrowths between the host bone and artificial bone (Moore et al. 2001).

Sadat-Shojai et al. (2013) has been review the HA as a material used for the medical applications. From the evaluation, they state that HA can be synthesize using several methods such as wet method, dry method, high temperature process method, biogenic sources method and combination procedure method. Each method may produced different structure and morphology of HA due to its different starting “Methods”. According to their study, focusing on the wet method, it consist of several sub-group methods, which is known as conventional wet chemical precipitation, hydrolysis method, sol–gel method, hydrothermal method, emulsion method and sonochemical method. The wet chemical precipitate method was selected in this research due to promise the HA product in nano size with regular morphology. According to the statistic, the conventional wet chemical precipitation method is usually used in synthesize HA due to the economical advantages and versatile route (Angelescu et al. 2011). Moreover, it is also one of the easy ways to prepared HA powder under atmospheric condition (Wang et al. 2010). However, it needs extra attention to control the Ca:P ratio as well as the crystallinity (Mustafa 2005). The physical and chemical properties of HA produce in this method is depend on the techniques used and calcium precursor sources. Thus, the different technique and calcium source can affect the thermal stability of the as synthesized HA produce (Kamalanathan et al. 2014).

Recently, the uses of wastes material in order to synthesize HA was received encourage response from many researcher across the world. This idea gives an innovation to produce a new valuable product from the wastes material. Besides that, these wastes material also can be recycled, then change it into more valuable things and keep environment safely In previous report, HA was synthesize by using wastes material such as sea shell (Santhosh and Prabu 2013), eggshell (Dávila et al. 2007), animal bones (Sobczak et al. 2009), shell of garden snail (Singh 2012), and fruit waste extract (Wu et al. 2013). These materials consist of high source of calcium that can be act as calcium precursor which is suitable to produce HA. Therefore, this study has focused on the synthesis of HA by using the ark clam shell (ACS) as wastes material to be used in synthesis HA via wet chemical precipitate method. Some researcher found that the content of calcium carbonate (CaCO_3_) in ACS is approximately between 98 and 99 % (Kamba et al. 2013; Mohamed et al. 2012). ACS which is rich in calcium content was seen pursuant act as an initial material in synthesize HA. Mustafa et al. (2015), synthesize HA from ACS by sol–gel precipitate method give a positive result in getting high purity of HA. Furthermore, Rujitanapanich et al. was successfully to synthesize HA from abalone shell which is same group of clam shell via the same method at various pH value from 8 to 10 (Rujitanapanich et al. 2014). They found that, the crystallinity of the HA from abalone shell is good compare to the HA from commercial CaO. From that, they observed the crystallize size of HA was in nano size about 89.5 nm. The previous researcher prove that the HA nanocrystalline can be prepared from waste material at room temperature by simple wet precipitate. In Table 1, the summarizes of recent studies in synthesis HA from waste material with various method and properties.

**Table 1:** Recent studies of synthesis HA powder from waste and various method in period of 2010–2015

References	Raw material	Method	pH value	Powder properties
Venkatesan and Kim (2010)	Tuna bone	Drying	–	Pure HA in nano-sized range
Wu et al. (2011)	Oyster shell	Ball mailing and heat treatment	–	HA and β-TCP composition in the sample
Singh and Purohit (2011)	Garden Snail	Wet chemical precipitate	10	Pure HA in micron-sized range
Santhosh and Prabu (2013)	Sea shell	Wet chemical precipitate	10	Pure HA in nano-sized range
Rujitanapanich et al. (2014)	Oyster shell	Wet chemical precipitate	8–10	Improve crystallinity of HA at pH value of 10.
Kamalanathan et al. (2014)	Eggshell	Wet chemical precipitate	10.5 and above	Pure HA at low temperature and present of α-TCP and TTCP at high temperature
Wu et al. (2015)	Eggshell	Wet chemical precipitate	–	HA/β-TCP biphase at low temperature and pure HA at high temperature
Chen et al. (2015)	Abalone shell	Solid-state conversion	10	Mix of HA, calcite and aragonite composition in the sample. HA nanorod is obtained
Yelmilda et al. (2015)	Cockle shell	Hydrothermal	10–11	Pure HA in nano-sized range and no toxic effect
Shavandi et al. (2015)	Mussel shell	Rapid microwave irradiation	13	Pure HA in nano-sized range
This study	Ark clam shell	Wet chemical precipitate	8	Pure HA as-synthesized and nano-sized range. HA/β-TCP biphase composition was present at high temperature

In this research, HA powder was synthesized via wet chemical precipitate method using ACS with control pH value at 8. The purpose of this research is to investigate the effect of HA on physical, chemical properties, morphology and behaviour of biphasic HA/β-TCP at various sintering temperature.

## Methods

ACS were thoroughly washed and cleaned to isolate the contaminate. Then, ACS was dried and transfer into the electrical furnace at a rate of 10 °C/min and holding at 900 °C for 4 h for the calcination process. The ACS consists of calcium carbonate (CaCO_3_) was transform to amorphous calcium oxide (CaO) by releasing carbon dioxide (CO_2_) during calcination process. The sample obtained was characterized by means of XRD to confirm the presence of CaO. Following Eq. (1) show the chemical reactions occur during calcinations process.

1 $${\text{CaCO}}_{3} \to {\text{CaO}} + {\text{CO}}_{2}$$

After that, the ACS has been through milling process with the milling balls in the jar at 100 rpm for 24 h to obtain a fine powder with a size of 45 μm. The obtained powder was used to prepare 1.0 M of calcium hydroxide solution (CaOH)_2_ by adding pre-determined amount of distilled water in the Eq. (2). The solution was stirred about 2 h to get the uniform mixing of (CaOH)_2_.

2 $${\text{CaO}} + {\text{H}}_{2} {\text{O}} \to {\text{Ca}}\left( {\text{OH}} \right)_{2}$$

In order to produce the HA powder, 0.6 M of phosphoric acid (H_3_PO_4_) was added to Ca(OH)_2_ at rate 15–20 drop/min by using titration technique with continuous stirring using a magnetic stirrer at room temperature. The estimated molar ratio of Ca:P was adjusted in the range between 1.67 and 2.00. The pH value of the solution was controlled and maintained around pH 8 by adding ammonium hydroxide solution (NH_4_OH) until the reaction was complete. The solution was continuously stirring and aging about 2 h. The gelatinous white precipitate was appeared when the solution was stop stirred. Subsequently, the solution was filtered and washed with distilled water several times before dried in the electrical furnace at 200 °C for 24 h to remove the water completely. The reaction of mixing of both solutions was showed below in Eq. (3) and the HA powder was produced.

3 $$10{\text{Ca}}\left( {\text{OH}} \right)_{2} + 6{\text{H}}_{3} {\text{PO}}_{4} {\text{Ca}}_{10} \to \left( {{\text{PO}}_{4} } \right)_{6} \left( {\text{OH}} \right)_{2} + 18{\text{H}}_{2} {\text{O}}$$

Then, HA powder was forms into pellet shape by using hydraulic press pellet machine with dimension of 13 mm in diameter and about 2 mm thick with 2.5 tons of applied load. Polyvinyl alcohol (PVA) were used as binder to avoid the pellet from crack and broken. The HA pellet were subjected to sintering in the furnace at temperatures of 200–1200 °C with an interval of 200 °C for 4 h and cooled in the furnace itself at room temperature. After the sintering process, the samples were characterized by XRD analysis using Philips X-ray diffractometer with Cu Kα radiation (λ = 1.5406 Å). The result was analyzed by using PANalytical X’Pert Pro PW3050/60 diffractometer at the diffraction angle (2θ) in scanning range from 20° to 60°. The densities of the samples were determined by using Archimedes method with water as the immersion liquid in this process. The molecular bonds structure of HA were observed by using FTIR(Perkim Elmer Spectrum 100 series) and FESEM (FEI NOVA NanoSEM 230) was used to study the morphology and microstructure of HA while EDX was carried out to analysis the elements present in HA.

## Result and discussion

### Characterization of ark clam shell to prepare hydroxyapatite

**Fig. 1:**
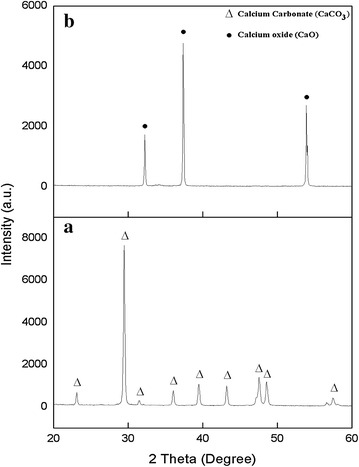
X-ray diffraction of the **a** ark clam shell and **b** calcined clam shell at 900 °C for 4 h

Physically, the ACS is tougher compared with calcined ACS and the appearance of calcined ACS produced was brittle and have yellowish white in colour. In detail, the XRD pattern of ACS in Fig. 1 shows the presence of pure CaCO_3_ phase while after treated the ACS at 900 °C calcination temperature, the pure CaO was observed by comparing with JCPDS files No. 005-0586 and 037-1497, respectively. The highest intensity peaks of CaCO_3_ were observed in the structure of ACS at 2θ = 29.5° while the highest peak of CaO was showed at a 2θ = 37.4° by calcined at 900 °C. In previous report, the decomposition of CaCO_3_ into the CaO and CO_2_ was occurs at the minimum temperature of 954 °C (Rujitanapanich et al. 2014). From XRD result, it can be observed that CaCO_3_ was completely converted to the CaO by calcination of CO_2_ at 900 °C for 4 h and eliminating other organic matter (Raya et al. 2015; Wang et al. 2007). The single phase of CaO also indicates that the complete transformation of CaCO_3_ to CaO has been occur. These results supported with previous reports, which suggest that the calcinations temperature of CaCO_3_ to CaO were at the range of 850–1000 °C due to the highest crystallinity and the formation of a single phase of CaO (Kamalanathan et al. 2014; Singh 2012). Singh (2012), mention that CaO is easily to be converted to Ca(OH) by absorbing moisture or water from surrounding atmosphere for the CaO prepared from shell of garden snail. Due to this reason, the precaution steps were taken by stored all the sample in desiccators to avoid vapor attack and taken out only during measurement of their properties. Besides that, the sample was preheated at 900 °C before use to remove the water in the sample.

**Fig. 2:**
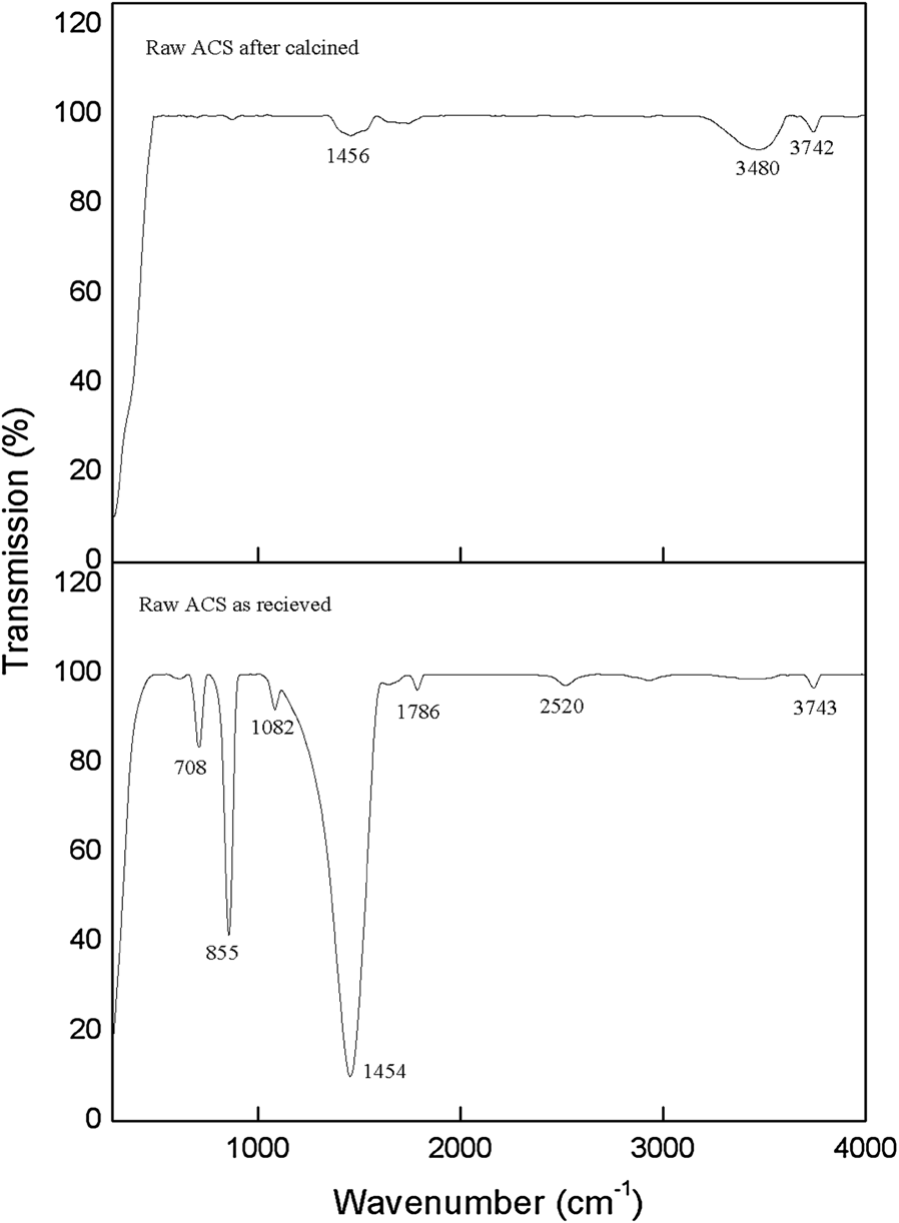
FTIR spectrum of ark clam shell and calcined ark clam shell at 900 °C for 4 h

Figure 2 shows the FTIR of ACS and calcined ACS at 900 °C in the frequency of 280–4000 cm^−1^. The ACS in Fig. 2a was further confirm contain of CaCO_3_ phase with significant characteristic peak at ~855 (v_2_), ~1454 (v_3_), and ~708 (v_4_) which indicate to the carbonate group in the sample. Besides that, the small infrared absorption spectra were shown at ~1786, ~2520, and ~2874 cm^−1^ attributed to the combination modes of different CO_3_^2−^ band (Kamalanathan et al. 2014; Gunasekaran et al. 2006). The spectra at ~3743 cm^−1^ are related to the stretching vibration of the hydroxyl group and C–O stretching mode was observed at ~1082 cm^−1^ as CO_2_ adsorbed on the surface of CaO (Kamalanathan et al. 2014). All detail of these modes and references is shown in Table 2.

**Table 2:** FTIR vibration modes of Clam shell powder and references

Assignments	Vibrational frequencies (cm^−1^)			
	FTIR result	Gunasekaran et al. (2006)	Islam et al. (2013)	Hoque et al. (2013)
*v*_4_—Symmetric CO_3_^2−^ deformation	708	712	706	705
*v*_2_—Asymmetric CO_3_^2−^ deformation	855	874	857	853
Symmetric stretching vibration of CO_3_^2−^	1082	–	1082	1080
*v*_3_—Asymmetric CO_3_^2−^ deformation	1454	1425	1455	1421
*v*_1_ + *v*_4_—CO_3_^2−^ deformation	1786	1798	1794	–
2*v*_2_ + *v*_4_—CO_3_^2−^ deformation	2520	2514	–	–
H_2_O stretching mode	3743	–	3378	–

The FTIR of ACS calcined at 900 °C is shown in Fig. 2b. The calcined ACS knows as CaO show the three small frequency band that corresponding to the structure of CaO. The frequency band at ~1454 cm^−1^ show the CO_3_^2−^ band become small compare to the spectra of ACS due to decomposition of CO_2_. The peak at ~3480 and ~3742 cm^−1^ is related to –OH bond group present in CaO. These result is supported from the previous report which stated that at ~3640 cm^−1^ is related to the O–H group (Galvan-Ruiz et al. 2007). The presence of moisture allow the CaO easily absorbed it and form of Ca(OH). As the result, the CaO sample cannot keep expose to environment in long time to avoid the CaO with moisture to become Ca(OH)_2_ (Loy et al. 2016). Due to this reaction, the result may affects the characteristic of CaO. The different pattern of FTIR spectra of both CaCO_3_ and CaO shown in Fig. 2 and the detail of frequency band of both was shown in Tables 2 and 3 respectively.

**Table 3:** FTIR vibration modes of calcined ark clam shell powder and references

Assignments	Vibrational frequencies (cm^−1^)			
	FTIR result	Singh and Purohit (2011)	Rujitanapanich et al. (2014)	Chen et al. (2015)
*v* C–O	1456	1422	1416	874, 1418, 1462
*v* O–H	3480	3431	–	–
*v* O–H	3742	–	3642	3567

### Characterization of hydroxyapatite (HA)

**Fig. 3:**
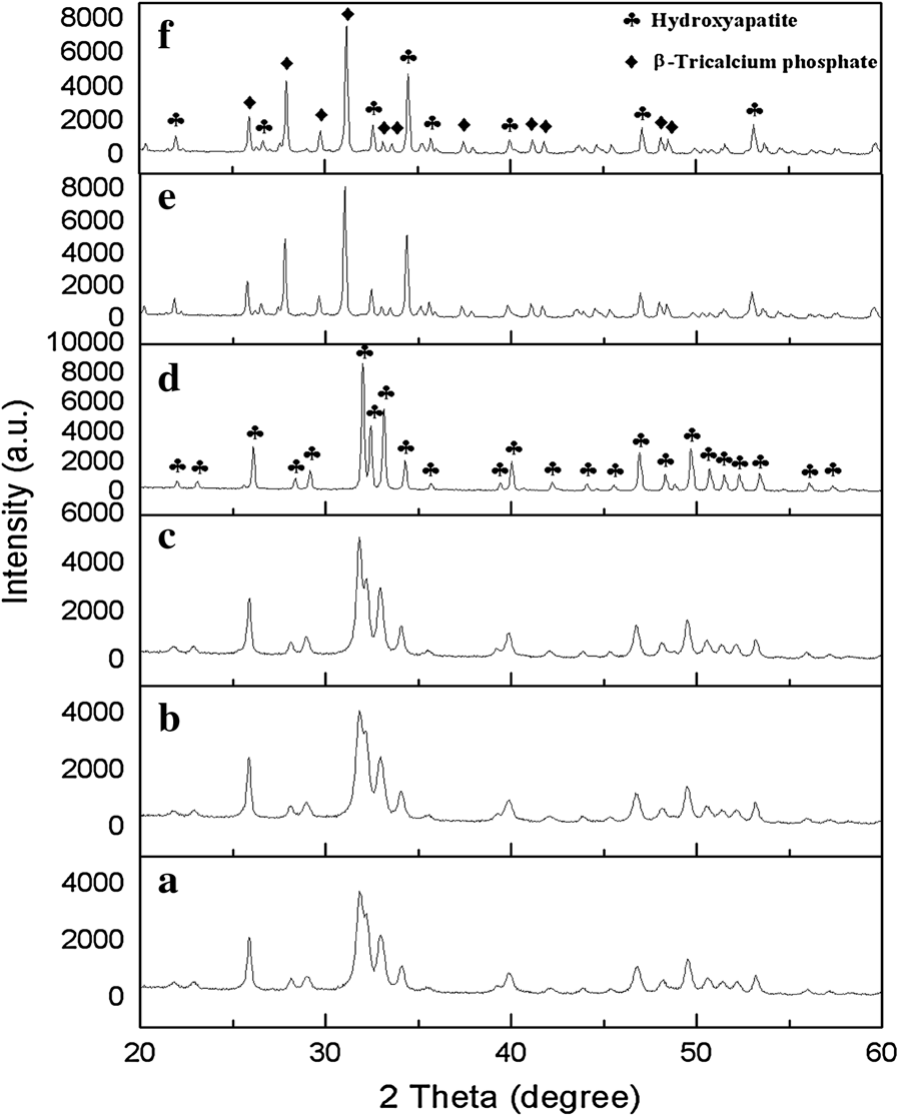
XRD patterns of sintered HA at different temperature for 4 h: **a** 200 °C, **b** 600 °C, **c** 800 °C, **d** 1000 °C, **e** 1100 °C and **f** 1200 °C

HA powder was analyzed by comparing with standard HA JCPDS file No. 001-084-1998 in range between 20° and 60°. The XRD pattern of HA sintered at 200–1000 °C (Fig. 3) was showed the single phase of HA only, without any secondary phase of HA decomposition. The secondary phases were consisting of another compound such as CaO, tricalcium phosphate (TCP) and tetracalcium phosphate (TTCP) (Singh 2012; Wu et al. 2013; Salma et al. 2010). Other researcher found the appearance of secondary phase due to the insufficient usage of either the calcium or phosphorous precursors (Kamalanathan et al. 2014). As precaution, the calculation of Ca:P ratio must in the range of 1.69–2.00 to obtained a single phase of HA in beginning synthesis process. From the result, the highest intensity peak detected at 2θ angle of 31.80° while the standard HA JCPDS is at 31.79° corresponding plane at (1 2 1). This highest peak indicates the HA was formed in the sample as resembled well with standard HA JCPDS file No. 001-084-1998. The peaks of intensity become more high and sharp as temperature increases from 200 to 1000 °C. These stability and purity of HA powder is very close to standard HA that used by the previous report (Venkatesan and Kim 2010). Unlike sintered HA powder at 1100 °C, HA was transform into new phase know as TCP as well as sintered at 1200 °C. The β-TCP phase was started become the dominant against HA in this stage. It is proving by comparing with standard β-TCP JCPDS file No. 000-009-0169 and presence of highest peak of β-TCP was detected at 2θ angle of 31.04°. In addition, the intensity of peaks of sintered HA was slightly decreases from at 1000 to 1200 °C.

**Fig. 4:**
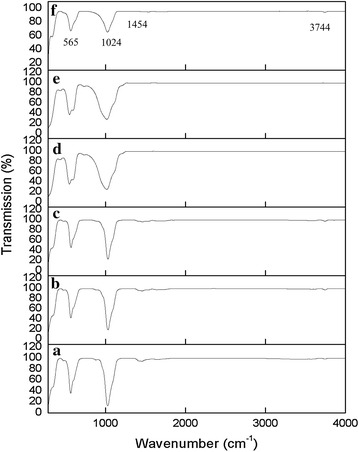
FTIR spectrum of sintered HA between 200 and 1200 °C for 4 h: **a** 200 °C, **b** 600 °C, **c** 800 °C, **d** 1000 °C, **e** 1100 °C, and **f** 1200 °C

Figure 4 shows the FTIR spectra of HA in the frequency between 280 and 4000 cm^−1^. The IR spectrum of HA consists of four absorption bands and it show the identical pattern and changes slightly during the temperature rise from 200 to 1200 °C for 4 h for each sample. The band was corresponding to the presence of PO_4_^3−^, CO_3_^2−^, and OH^−^ in the sample. The frequency of ~565 cm^−1^ was detected corresponding to PO_4_^3−^ symmetric bending mode (*v*_4_) while at ~1024 cm^−1^ was corresponding to PO_4_^3−^ asymmetric stretching mode (*v*_3_). Both PO_4_^3−^ mode (*v*_3_) and PO_4_^−3^ mode (*v*_4_) corresponding to the vibrational groups to the structure of HA. In addition, the band positioned at ~1454 cm^−1^ is indicating to the CO_3_^2−^ group (*v*_3_). Structure of OH^−^ vibrational mode was observed only at low temperature at 3744 cm^−1^ and totally not visible at 1100 °C. Table 4 shows the references of HA FTIR vibration modes sintered at various temperature.

**Table 4:** FTIR vibration modes of HA sintered at 200 °C and references

Assignments	Vibrational frequencies (cm^−1^)			
	FTIR result	Salma et al. (2010)	Singh and Purohit (2011)	Kamalanathan et al. (2014)
PO_4_^3−^ bending *v*_4_	565	560, 599	567	568
PO_4_^3−^ stretching *v*_3_	1024	1046	1046	1038
CO_3_^2−^ group *v*_3_	1454	1424, 1424	1422	1421
OH^−^ structure	3744	1637, 3100–3700	3431	3425

### Crystallite size and density of hydroxyapatite (HA)

The sintering temperature of HA was affect the physical of the sample. Generally, as the sintering temperature increases, the size of HA pallet and porosity will decreases due to the grains shifting to more dense packing. Table 4 showed the crystallite size, linear linkage and density at various sintering temperature of HA. From the result, it can be described that as temperature increases, the crystallite size of HA become increases (Abidi and Murtaza 2014). From the previous reports, the researchers have proved the high temperature of HA sintered show the improvement in the high peak of crystallinity (Abidi and Murtaza 2014; Bouyer et al. 2000). It is reported that samples with good crystallinity show little or no activity in the bioresorption activity, which is important for formation of chemical bonding with hard tissue area (Sanosh et al. 2009; Aoki 1994). Thus, the amorphous HA were obtained at lower temperature are expected to be more metabolically active rather than high temperature of HA which show the fully developed crystalline HA structure which otherwise insolubility in physiological environment (Sanosh et al. 2009; Kim et al. 2000).

As can be seen in the Table 5, it is expected the linear shrinkage increases as the sintering temperature increases from 200 to 1200 °C. The increasing in shrinkage of the sample was due to the eliminating of porosity or pore in the sample as sintering temperature increases (Mustafa 2005; Kamalanathan et al. 2014; Champion 2013; Ramesh et al. 2008). Besides that, the density of HA increases with increasing the sintering temperature from 2.76 to 3.12 g/cm^3^ at 200 to 1000 °C respectively can be confirm due to the porosity decreases and grains of HA shift to obtain more dense packing particle (Kamalanathan et al. 2014). However, at high temperature of 1000–1200 °C represent the decrement of density value from 3.12 to 2.86 g/cm^3^. The decreasing density value from 1000 to 1200 °C may due to partially decomposition of HA phases to TCP phases (Mustafa 2005; Kamalanathan et al. 2014; Hung et al. 2012; Mobasherpour et al. 2007; Raynaud et al. 2002).

**Table 5:** The crystallite size and linear shrinkage of the HA sample at various sintering temperature

Temperature (°C)	Crystallite size [D (nm)]	Linear shrinkage (%)	Density (g/cm^3^)
200	26.58	0	2.76
600	29.54	3.08	2.81
800	37.98	6.15	2.96
1000	53.18	11.53	3.12
1100	66.32	14.84	2.93
1200	66.34	16.69	2.86

### Microstructure of hydroxyapatite (HA) FESEM and EDX

**Fig. 5:**
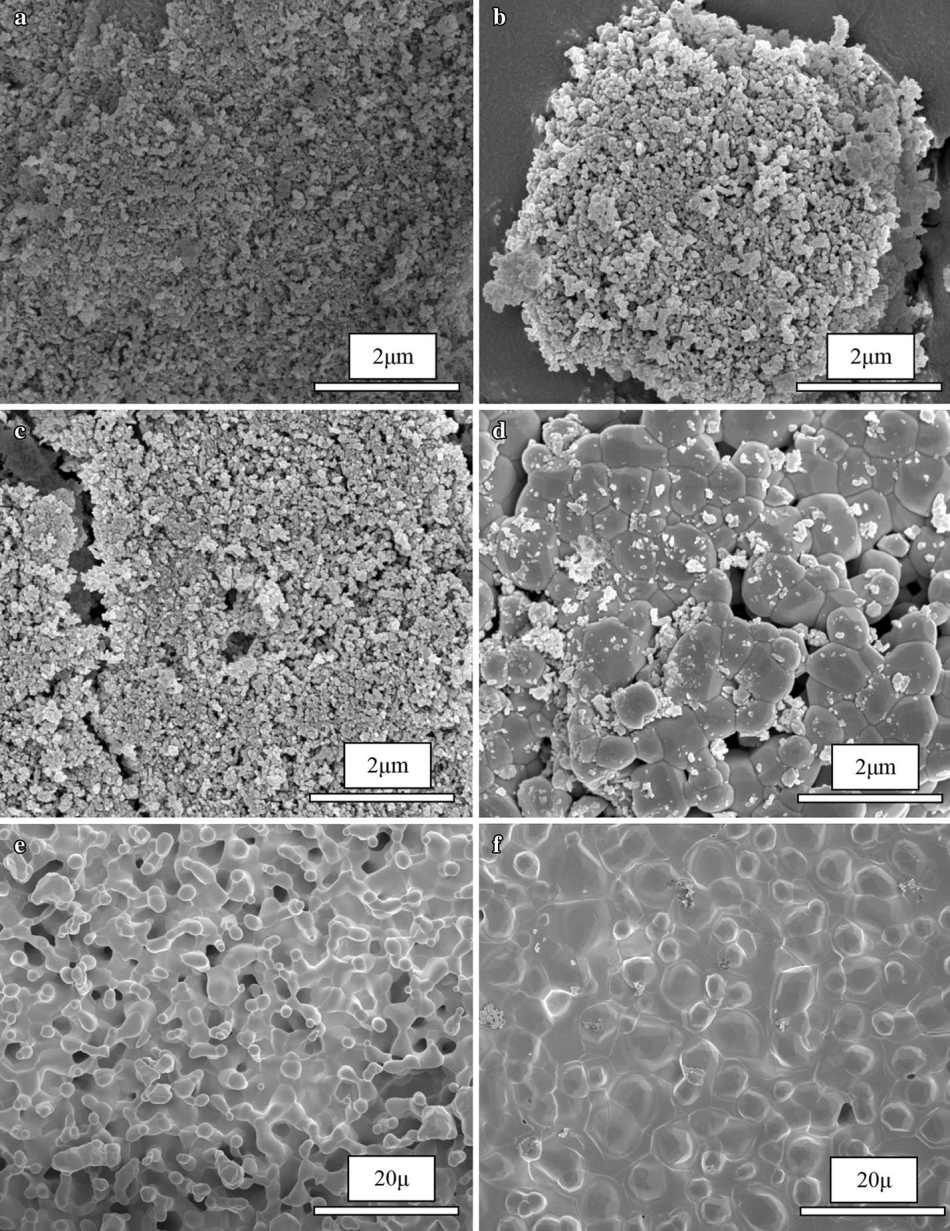
FESEM micrograph of HA: **a** 200 °C, **b** 600 °C, **c** 800 °C, and **d** 1000 °C under 50 k magnification and **e** 1100 °C and **f** 1200 °C under 5 k magnification

Figure 5 show the FESEM images of the sintered HA from 200 to 1200 °C under Ca:P ratio below of 2.00 and pH value of 8. Its can clearly observed the stage of evolution microstructure of sintered HA samples. The particle size is found to be increases parallel as increasing the sintering temperature of the samples. At temperature 200–800 °C, the irregular shape of particles was observed. Besides that, the microstructure of HA are mostly agglomerated in this stage. This strong agglomerated was occurring because of the presence of water molecule that could not remove from the sample by freeze drying process (Yoruc and Koca 2009). The presence of HA nanoparticle size was observed at 200 °C in between ~35 and ~69 nm in Fig. 5a. The further sintering temperature in Fig. 5b, c was show the increases of the HA particle size at 600 and 800 °C was recorded between ~0.13–0.24 and ~0.33–0.79 μm respectively. As sintering temperature increases from 1000 to 1200 °C, the morphology of HA become increases in grain size. At 1000 °C, morphology of HA start become granular in shape and irregular grain of HA was observed. Further sintering temperature at 1100 °C, the single phase of HA decomposed to a new phase as HA/β-TCP phase. The necking among particles becomes more prominent in this temperature due to high temperature sintering. The dense grain become significant at 1200 °C and it clearly observed the shape exhibited denser packing grain and the porosity is decreases due to pore removing occurs during densification. The increases of sintering temperature help the sample to improve the homogeneity and grain growth resulting in an increase in mean grain size (Mustafa 2005). In the Fig. 5d–f show the HA particle size was obviously increased parallel to the sintering temperature (1000, 1100 and 1200 °C) is between ~0.34–0.79, ~1.96–4.68, and ~1.87–6.40 μm respectively.

**Fig. 6:**
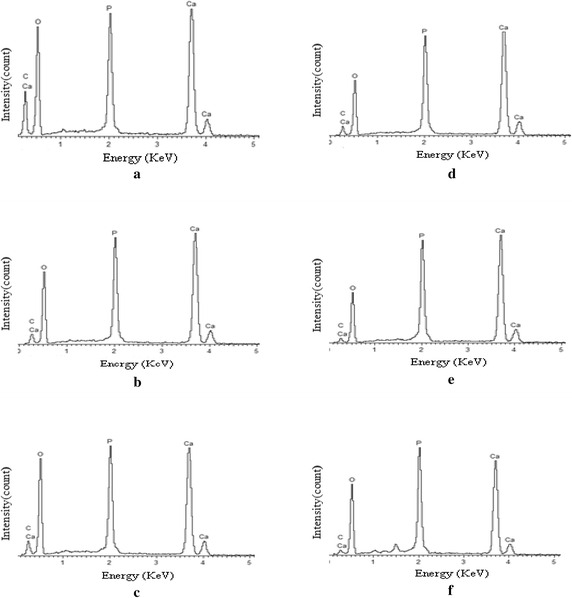
EDX spectra of HA at different sintering temperatures: **a** 200 °C, **b** 600 °C, **c** 800 °C, **d** 1000 °C, **e** 1100 °C, and **f** 1200 °C

EDX analysis are used for determine the element or chemical composition of the HA which is different in sintering temperature. Figure 6 represent the EDX spectra of the entire sample sintered at various temperatures from 200 to 1200 °C. From the result, the presence of element such as Ca, P, C, and O are observed and the Ca:P ratio are calculated in Table 6 to compare with the theoretical ratio which is 1.67. The element composition in the HA sample with different sintering temperature was summarized in Table 6. From the result, the Ca:P ratio of HA decreases as the sintering temperature increases. Although that, at temperature of 1000 °C, the Ca:P ratio immediately increases and it decreases back at temperature of 1100 °C. The pattern continues to decreases until the temperature at 1200 °C which is calculated the value of Ca:P ratio is 1.66. From overall result of EDX spectra, the calculated Ca:P ratio of HA decreases from 1.95 to 1.66 as HA undergo sintering process for 4 h with each sintering temperature. In detail, as the sample of HA were sinter at high temperature, there was a possibility for the HA decompose to biphasic in form of HA/TPC (Mustafa 2005; Kamalanathan et al. 2014; Hung et al. 2012; Mobasherpour et al. 2007; Raynaud et al. 2002). The presence of oxygen in EDX spectra shows the decreases in the element weight percentage from 200 to 1100 °C. However, at temperature of 1100 °C, the oxygen was started to rise in the element weight percentage of due to the present of possibility of CaO or β-TCP as another phase in the sample. From the previous research, they found the existence of CaO phase in the HA when the Ca:P ratio was high than 1.67 (Abidi and Murtaza 2014; Best et al. 2008).

**Table 6:** The element composition present in the HA sample with different sintering temperature

Sintering temperature (°C)	Weight of element composition (wt%)				Ca:P ratio
	Ca	P	C	O	
200	25.64	13.12	17.48	43.76	1.95
600	31.61	16.61	7.05	44.74	1.90
800	27.83	15.27	8.20	48.70	1.82
1000	34.45	17.35	7.27	40.92	1.98
1100	37.06	18.76	3.78	40.40	1.97
1200	30.96	18.63	3.41	47.00	1.66

## Conclusion

The usability of ACS as a CaO source to produced hydroxyapatite via wet chemical precipitated method was characterize by XRD, FTIR, FESEM and EDX results. The using waste materials can cut off the cost of synthesis material since the quality and purities of the product almost same as commercial HA. In this studies, the formation of as-synthesize HA nanoparticle (~35–69 nm) through using waste precursor resources was successful done. The characterization of HA depend on various parameter such that Ca:P ratio, pH value and the temperature of initial solution. From this research, the some of the HA phase is start decompose to β-TCP phase at temperature of 1100 °C with fix pH value at 8 and the Ca:P ratio value above 1.67 but was control below 2. The microstructure and morphology of HA show the relationship of temperature. As the sintering temperature increases, HA particle was forming a dense particle and resulted in increasing density of HA. It was confirmed that the density decreases due reduced in porosity in the sample by increase the sintering temperature.
